# A low-cost and high-performance thin-film composite forward osmosis membrane based on an SPSU/PVC substrate

**DOI:** 10.1038/s41598-018-28436-4

**Published:** 2018-07-03

**Authors:** Ke Zheng, Shaoqi Zhou, Xuan Zhou

**Affiliations:** 1 School of Environment and Energy, South China University of Technology, Guangzhou Higher Education Mega Center, Guangzhou, 510006 P. R. China; 2 Guizhou Academy of Sciences, Shanxi Road 1, Guiyang, 550001 P. R. China; 3 State Key Laboratory of Subtropical Building Science, South China University of Technology, Guangzhou, 510641 P. R. China; 4 The Key Lab of Pollution Control and Ecosystem Restoration in Industry Clusters, Ministry of Education, South China University of Technology, Guangzhou Higher Education Mega Centre, Guangzhou, 510006 P. R. China

## Abstract

A low-cost sulfonated polysulfone (SPSU)/poly(vinyl chloride) (PVC) substrate based high-performance thin-film composite (TFC) forward osmosis (FO) membrane was fabricated in this work. The results showed that the morphologies of the substrates were looser and more porous, and the porosity, pure water permeability, surface hydrophilicity, and average pore size of the substrates significantly improved after the SPSU was introduced into the PVC substrates. Furthermore, the SPSU/PVC-based TFC membranes exhibited rougher, looser and less crosslinked polyamide active layers than the neat PVC-based TFC membrane. The water permeability obviously increased, and the structure parameter dramatically declined. Moreover, the FO performance significantly improved (e.g. the water flux of TFC2.5 reached 25.53/48.37 LMH under FO/PRO mode by using 1.0 M NaCl/DI water as the draw/feed solution, while the specific salt flux exhibited a low value of 0.10/0.09 g/L). According to the results, it can be concluded that 2.5% of SPSU was the optimal blend ratio, which exhibited the lowest sulfonated material blend ratio compared to the data reported in the literature. Hence, this is a feasible and low-cost fabrication approach for high-performance FO membrane by using the cheap PVC and low blend-ratio SPSU as the membrane materials.

## Introduction

Forward osmosis (FO) has attracted the attention of researchers as an energy-efficient membrane technology in recent years^1–4^. Without requiring a high hydraulic pressure, FO utilizes osmotic pressure differences to drive the water across the FO membrane from a low-osmotic-pressure feed solution to a high-osmotic-pressure draw solution^2^. Hence, compared to traditional pressure-driven separation technologies, such as reverse osmosis (RO) and nanofiltration (NF), FO has several merits, including: 1) lower energy consumption and equipment costs; 2) less membrane fouling; 3) higher water recovery; and 4) wider applications^5–8^. However, FO membranes have a critical problem, internal concentration polarization (ICP). The ICP can be ascribed to the resistance to diffusion of the FO membranes, which leads to a reduction in the osmotic pressure across the membrane^9^. Compared to the external concentration polarization (ECP), which occurs outside the FO membranes, the ICP resides in the membranes, which means that it cannot be alleviated by hydrodynamics optimization^9^. Hence, FO membrane fabrication optimization is a practical method to relieve the ICP.

Fabrication of high-performance FO membranes is a research hotspot in the FO field^10–13^. An ideal FO membrane should have a high water flux and limited reverse salt flux during a FO process^14^. Generally, FO membranes can be divided into three types: (1) asymmetric membranes^15,16^; (2) layer-by-layer self-assembled membranes^17,18^; and (3) thin-film composite (TFC) membrane^8–11^. Because of their good FO performance and wide pH application range, TFC FO membranes have gained the most attention from FO researchers. The TFC FO membrane consists of a porous substrate layer and an ultra-thin active layer^19^. Moreover, the substrate plays an important role in the performance of FO membranes. Hence, most researchers of TFC FO membranes focus on substrate studies. Polysulfone (PSU), polyethersulfone (PES) and polyvinylidene fluoride (PVDF) are the most popular backbone materials for preparing the substrates for TFC FO membranes^3^. However, these materials are hydrophobic polymers. Hence, the hydrophilicity of the substrate needs to be improved to decrease the ICP and improve the performance. Introduction of hydrophilic polymers and nanoparticles into substrates are the feasible strategies^3,14^. For instance, Zhou *et al*.^9^ modified a PSU substrate by blending it with sulfonated poly(phenylene oxide) (SPPO). Their work demonstrated that the introduction of SPPO significantly improved the performance of the TFC FO membrane. Emadzadeh *et al*.^19^ introduced TiO_2_ nanoparticles into the substrate. Their results illustrated that the porosity and hydrophilicity obviously improved after the modification, which led to a considerable remission of the ICP. Wang and Xu^12^ blended PES, sulfonated polyethersulfone (SPES) and montmorillonite to fabricate the substrate. Their results illustrated that montmorillonite could anchor the SPES in the substrate to improve the FO performance.

Generally, backbone materials and hydrophilic modifiers contribute to the costs of membranes^20,21^. The widely used backbone materials (e.g. PSU, PES and PVDF) are not low-cost products, and the modifier materials are even more expensive or lab-made products. Hence, the manufacturing costs of FO membranes are an obstacle for the application of the FO technology. Using a low-cost backbone material and reducing modifier use could lower the manufacturing costs. Poly(vinyl chloride) (PVC), which is one of the most widely-used and low-cost resins (<1/10 price of PSU, PES, or PVDF), exhibits excellent characteristics of acid and alkali resistance, abrasion resistance and good mechanical strength^20^. Moreover, PVC can be dissolved in various industrial solvents^22^. Hence, PVC can be applied for the fabrication of membranes via the nonsolvent-induce phase separation (NIPS) process^20,22–24^. However, due to its inherent hydrophobicity, hydrophilic modification is necessary for fabricating PVC membranes. On the other hand, sulfonated polymers, such as SPPO^9^, SPES^12^, sulfonated polysulfone (SPSU)^25^, sulfonated poly(ether ketone) (SPEK)^26^, disulfonated poly(arylene ether sulfone) (DSPAES)^27^, and polyethersulfone and sulfonated polyphenylsulfone copolymer (PES-co-SPPSU)^28^, are widely used to improve the hydrophilicity of membranes. However, a high blend ratio of the sulfonated polymers is unfavorable for cost control. Hence, a reasonable blend ratio of sulfonated polymers should be required and studied.

This work aims to fabricate a high-performance TFC FO membrane with low manufacturing costs. Therefore, PVC was chosen as the low-cost backbone material and SPSU as the hydrophilic modifier material for preparation of the substrates for the TFC FO membranes in this study. The SPSU/PVC substrates were first prepared for fabrication of the TFC FO membranes. The characteristics and performances of a series of SPSU/PVC substrates were fully investigated, including the morphology (by SEM study), porosity, pure water permeability (PWP), surface hydrophilicity (by contact angle), and average pore size. Moreover, the effect of the properties of the SPSU/PVC substrates on the morphology and intrinsic properties of the TFC FO membranes was investigated. Finally, the optimal blend ratio of SPSU and PVC was determined through the intrinsic properties and FO performance of the TFC membranes.

## Materials and Methods

### Chemicals and membrane materials

PVC (P440, k-value of 73–75, Shenfeng Chemicals Co. Ltd., China) and SPSU (sulfonation degree = 20%, Shanghai Yunli Polymer Co. Ltd., China) were used as the materials for preparation of the substrates. 1-Methyl-2-pyrrolidinone (NMP, >99.5%, Aladdin) was used as the solvent for preparation of the casting solutions. M-phenylenediamine (MPD, >99%, Sigma-Aldrich), trimesoyl chloride (TMC, >98%, Sigma-Aldrich) and *n*-hexane (>99%, Aladdin) with were used for the interfacial polymerization (IP) process. Sodium chloride (NaCl, >99.5%, Aladdin) was used as the draw solute.

### Fabrication of flat-sheet TFC FO membranes

#### Preparation of blended substrates via phase inversion

The preparation of the SPSU/PVC substrates was based on the NIPS process. The casting solution compositions are provided in Table 1. PVC and SPSU were dissolved in NMP and continuously stirred at 60 °C for 24 h to obtain a homogeneous and transparent solution. After degassing at room temperature for 24 h, the casting solution was cast on a pre-cleaned glass plate using a 150 μm stainless-steel casting knife. After evaporation in a fume hood for 30 s, the as-cast substrates with the glass plate were immersed into a deionized (DI) water coagulation bath at the ambient temperature to initiate the phase inversion. Afterwards, the obtained substrates were transferred into a flowing DI water bath for 48 h to remove residual solvent before further use.

**Table 1:** Composition of the casting solutions for the preparation of the blended substrates.

Membranes	Total polymers (wt%)	PVC (wt%)	SPSU (wt%)	NMP (wt%)	m(SPSU):m(PVC)
S0	15	15.000	0.000	85	0.0: 100.0
S0.5	15	14.925	0.075	85	0.5: 99.5
S1	15	14.850	0.150	85	1.0: 99.0
S2.5	15	14.625	0.375	85	2.5: 97.5
S5	15	14.250	0.750	85	5.0: 95.0
S10	15	13.500	1.500	85	10.0: 90.0

#### Preparation of the polyamide active layer

The polyamide (PA) active layer was prepared with MPD and TMC monomers via an interfacial polymerization (IP) process on the surface of a neat PVC or SPSU/PVC substrate. First, the prepared substrate was immersed in an aqueous solution of 2.0 wt.% for 120 s. The excess MPD solution was removed by pure compressed nitrogen gas for 2 min. Then, a 0.1 wt.% *n*-hexane solution of TMC was gently poured onto the MPD-soaked substrate surface to form the PA layer for 60 s. After the *n*-hexane solution was drained off, the nascent TFC membrane was air-cured for 5 min. The resultant TFC membrane was rinsed with DI water to remove the residual monomers and then stored in DI water before characterization and performance testing. These membranes are denoted as TFC0, TFC0.5, TFC1, TFC2.5, TFC5, and TFC10 according to the label names of the substrates.

### Light transmittance experiment

Light transmittance experiments were performed using a self-made device, and more details were provided in Yu’s study^29^. The casting solution was cast on a glass plate with the same procedure of preparation of the substrates. The glass plate was immersed into the DI water bath used as the coagulation fluid. The light source was directly above the casting solution, approximately 30 cm. An optical detector (DT1309, Huashengchang, China) was used to detect the transmitted light, and the detected data were recorded by a computer.

### Membrane characterization

#### Morphology of the substrates and active layer

The morphology of the substrate and active layer was observed with a high-resolution field emission scanning electron microscope (HR-FESEM, Merlin, Carl Zeiss, Germany). The samples of the substrates and TFC FO membranes were first freeze dried for 48 h. The samples were coated with platinum by a sputtering coater (150 T, EMS, UK). The samples were flash-frozen and cracked in liquid nitrogen for observation of the cross-section before freeze drying.

#### Properties of the substrates and active layer

The PWP of the substrates was determined in a lab-scale cross-flow filtration device, which gives an effective membrane area of 11.34 cm^2^. All substrates were pre-compacted at 1.5 bar for 60 min to obtain a steady flux. The PWP data were tested at 1.0 bar after the pre-compaction.

The porosity (*ε*) of the substrate was based on the difference between the wet and dry weights of the substrate sample by Eq. (1), where *m*_1_, *m*_2_, *ρ*, *T* and *A*_*m*_ represent the wet weight, dry weight, the density of DI water, thickness and effective area of the substrate sample, respectively.

1 $$\varepsilon =\frac{{m}_{1}-{m}_{2}}{\rho \times T\times {A}_{m}}$$

The average pore size (*r*_*m*_) was calculated based on the PWP and porosity data by using the Gerout-Elford-Ferry equation (Eq. (2)), where *η* and *T* represent the viscosity of DI water and thickness of the substrate sample, respectively.

2 $${r}_{m}=\sqrt{8\times \frac{(2.9-1.75\varepsilon )\times \eta \times T\times PWP}{\varepsilon }}$$

The contact angle of the substrate was measured by a contact angle goniometer (DSA25, Kruss, Germany). Droplets of DI water (2 μL) were applied onto a pre-dried substrate surface to test the contact angle. Five measurements were carried out at random locations on a substrate sample.

The chemical composition of the polyamide active layer was analyzed by X-ray photoelectron spectroscopy(XPS, Escalab 250xi, Thermo Fisher Scientific, UK) using a monochromatic Al X-ray source.

#### Intrinsic properties of the TFC membrane

The water permeability (*A*) and salt permeability (*B*) were determined in the same filtration device applied to test the PWP of the substrates. The *A* value was measured using DI water as the feed solution under a pressure of 5.0 bar. The *A* value was calculated using Eq. (3), where Δ*V*_*a*_ is the permeate volume in the water permeability test over a fixed time Δ*t*_*a*_, *A*_*m*_ is the effective area of the TFC membrane, and Δ*P* is the transmembrane pressure difference.

3 $$A=\frac{{\rm{\Delta }}{V}_{a}}{{\rm{\Delta }}{t}_{a}\times {A}_{m}\times {\rm{\Delta }}P}$$

The salt permeability (*B*) was calculated based on the salt rejection (*R*_*s*_) and *A* value^19^. The *R*_*s*_ value was measured by using a 2000 ppm NaCl solution as the feed solution under a pressure of 5.0 bar. The *R*_*s*_ and *B* values were calculated by Eqs (4) and (5), respectively, where *C*_*f*_ and *C*_*p*_ are the NaCl concentrations of the feed and permeate solutions and Δ*P* and Δ*π* are the transmembrane hydraulic and osmotic pressure differences, respectively.

4 $$RS=\frac{{C}_{f}-{C}_{p}}{{C}_{f}}\times 100 \% $$

5 $$B=A\times (\frac{1}{{R}_{S}}-1)\times ({\rm{\Delta }}P-{\rm{\Delta }}\pi )$$

The structure parameter (*S*) can be estimated by the classical flux-fitting method with Eq. (6)^30,31^, where *D* is the solute diffusion coefficient, *π*_*draw*_ and *π*_*feed*_ are the osmotic pressures of the draw solution and feed solution, respectively, and *J*_*W*_ is the water flux under FO mode in the performance test which would be discussed in section of FO performance tests.

6 $$S=\frac{D}{{J}_{W}}[\mathrm{ln}\,\frac{A\times {\pi }_{{draw}}+B}{A\times {\pi }_{{feed}}+{J}_{W}+B}]$$

### FO performance tests

The FO performance was evaluated on a lab-scale cross-flow filtration setup with an effective membrane area of 12 cm^2^ (2 cm × 6 cm). The feed and draw solutions were circulated co-currently through rectangular channels at a fixed crossflow rate of 480 mL/min. The DI water and NaCl solution were used as feed and draw solutions, respectively. The weight changes in the NaCl solution were recorded by a data-logging balance (AX4202ZH, Ohaus, USA) to reflect the water flux (*J*_*W*_), and the NaCl concentration changes in the feed solution were recorded by a conductivity meter (DDSJ-308F, Rex, China) to reflect the reverse salt flux (*J*_*S*_). All of the FO performance tests were carried out for 75 min, and the *J*_*W*_ and *J*_*S*_ values were calculated by the average of the data from the last 60 min. Particularly, the FO performance was tested under two operation modes, namely, FO mode, where the active layer faced the feed solution, and PRO (pressure-retarded osmosis) mode, where the active layer faced the draw solution.

The *J*_*W*_ and *J*_*S*_ values were calculated by Eqs (7) and (8), where Δ*V* is the volume change of the DS over a fixed time (Δ*t*), *S*_*m*_ is the effective area of the TFC membrane, *C*_*t*_ and *C*_0_ are the initial and final concentrations of the feed solution, respectively, and *V*_*t*_ and *V*_0_ are the initial and final volumes of the feed solution.

7 $${J}_{W}=\frac{{\rm{\Delta }}V}{{S}_{m}\times {\rm{\Delta }}t}$$

8 $${J}_{S}=\frac{{C}_{t}\times {V}_{t}-{C}_{{0}}\times {V}_{{0}}}{{S}_{m}\times {\rm{\Delta }}t}$$

### Data availability statement

The data of this study are available from the corresponding author on reasonable request.

## Results and Discussion

### Phase inversion properties

**Figure 1:**
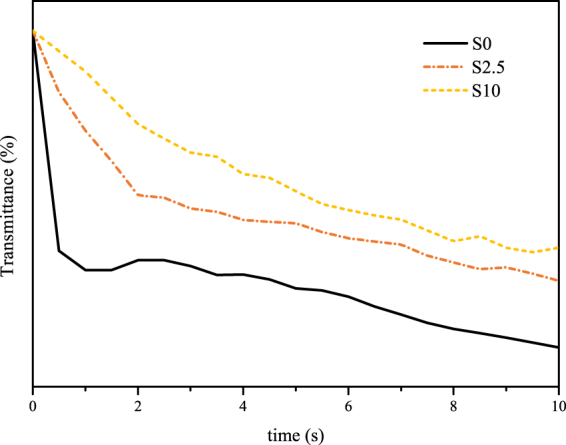
Light transmission test of the casting solution.

Light transmission experiments were carried out to illustrate the effect of the blend ratios of SPSU and PVC on the phase inversion mechanism of the substrates. The phase inversion process will lead to the optical inhomogeneity of the casting solution and decrease the light transmittance. Therefore, the decreasing rate of light transmittance can be used to represent the rate of the phase inversion process^32^. Figure 1 shows the results of the light transmission experiments. The phase inversion speed became slower after SPSU was introduced into the casting solution. This phenomenon may arise from the presence of sulfonic acid groups in SPSU, which can form hydrogen bonds with water molecules. Hydrogen bonding can enhance the water tolerance of the SPSU-introduced casting solutions and delay the solvent (NMP) and the non-solvent (water) exchange^33^. Obviously, the introduction of SPSU affected the phase inversion process, which will have an important effect on the morphology and performance of the prepared substrates.

### Characteristics and performances of the substrates

#### Morphology of the substrates

**Figure 2:**
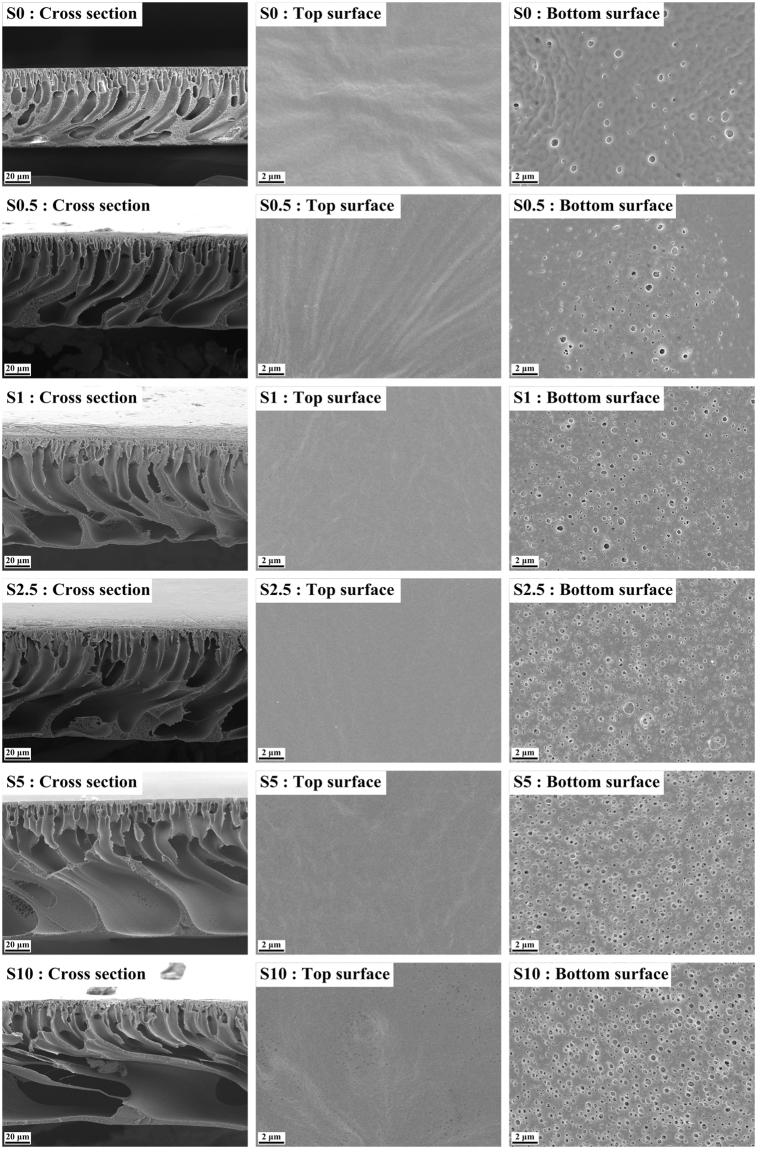
FESEM images of the substrates with different SPSU/PVC blend ratio.

Figure 2 shows the FESEM images of the cross-sections, top surfaces, and bottom surfaces of the substrates. According to the cross-section images, all the substrates exhibited typical asymmetric morphologies. However, the increasing SPSU/PVC blend ratio led to a noticeable difference in the morphology of the substrates. For the neat PVC substrate, numerous “lanky” finger-like pores were separated by a the sponge-like medium in between. After the SPSU was introduced into the substrates, the finger-like pores became larger and more irregular significantly. Eventually, the pores became interconnected near the bottom part of the substrates at the SPSU/PVC blend ratio of 10%. This means that the SPSU is beneficial for improving the porosity of the substrates, which would be confirmed by the porosity test results. This morphology change should come from the slower phase inversion speed. According to Blanco’s theory^34^, the slower the phase inversion rate, the more developed the polymer-lean phase growth and coalescence, resulting in the larger finger-like pores, and a similar phenomenon was found in Ren’s research^33^. It is believed that the looser and more porous substrates could conduce to decline the ICP in the FO process^19^. Moreover, it can be seen in the cross-section images that the thicknesses of the substrates increased after the SPSU was introduced, and the increment of the thickness should be related to the increase in the thermodynamics instability caused by SPSU^35^. Similar phenomena were found in Ou^36^ and Wang’s^12^ works.

According to Fig. 2, the top surface images exhibited few differences for all substrates. All the top surfaces exhibited dense surface morphologies with a few small pores. However, compared to the top surfaces, the bottom surfaces of all the substrates exhibited more open and porous morphologies with larger pores. Along with the increase in the SPSU/PVC blend ratios, the bottom surfaces became significantly more porous. The more open bottom morphology could accelerate the salt diffusion from the draw solution into the substrate (under FO mode) to alleviate the dilutive ICP, or from the substrate to the feed solution (under PRO mode) to alleviate the concentrative ICP^9^.

#### Properties of the substrates

Table 2 summarizes the properties of the substrates prepared with different SPSU/PVC blend ratios. The hydrophilicity of the substrates was illustrated by contact angel tests. The contact angle value declined from 84.9° for S0 to 75.5° for S10. This result indicates that the introduction of SPSU enhanced the hydrophilicity of the substrates significantly due to the presence of the sulfonic acid groups on the SPSU. Moreover, the overall porosity (*ε*) and the mean pore size (*r*_*m*_) increased with the increase in the SPSU/PVC ratio. The major reason of this phenomenon can be ascribed to the larger size of the finger-like pores caused by the lower phase inversion rate^33,34^. An obvious improvement in the porosity of the S0.5 (86.0%) was observed compared to that of S0 (81.1%). Moreover, the porosity slightly improved to 90.2% for S10. It is believed that the higher value of the porosity is the main factor contributing the smaller structure parameter (*S* value) of the substrate, which leads to a smaller ICP during a FO process^19^. Furthermore, the PWP got obviously improved after the SPSU was introduced. According to the Hagen-Poiseuille pore flow model, the increasing mean pore size and porosity should be ascribed to a crucial factor in the improvement of the PWP^37^. The hydrophilicity of the SPSU could improve the water permeability by drawing water molecules into the substrate and facilitating their transportation throughout the substrate^36^.

**Table 2:** Summary of the SPSU/PVC substrates characteristics.

Membranes	*ε* (%)	PWP (LHM/bar)	Contact angle (°)	*r*_*m*_ (nm)
S0	81.1 ± 0.6	54.1 ± 4.2	84.9 ± 0.8	12.8 ± 0.8
S0.5	86.0 ± 0.2	77.7 ± 1.6	79.9 ± 1.6	15.1 ± 0.3
S1	87.7 ± 0.1	121.3 ± 8.4	78.2 ± 1.2	20.2 ± 0.9
S2.5	89.1 ± 0.4	208.8 ± 11.0	77.1 ± 1.2	26.6 ± 0.5
S5	89.5 ± 0.3	315.5 ± 25.1	76.7 ± 2.1	34.4 ± 1.5
S10	90.2 ± 0.2	395.5 ± 32.5	75.5 ± 2.7	37.9 ± 0.1

### Characteristics and performances of the TFC membranes

#### Morphology and properties of the active layer of the TFC membrane

**Figure 3:**
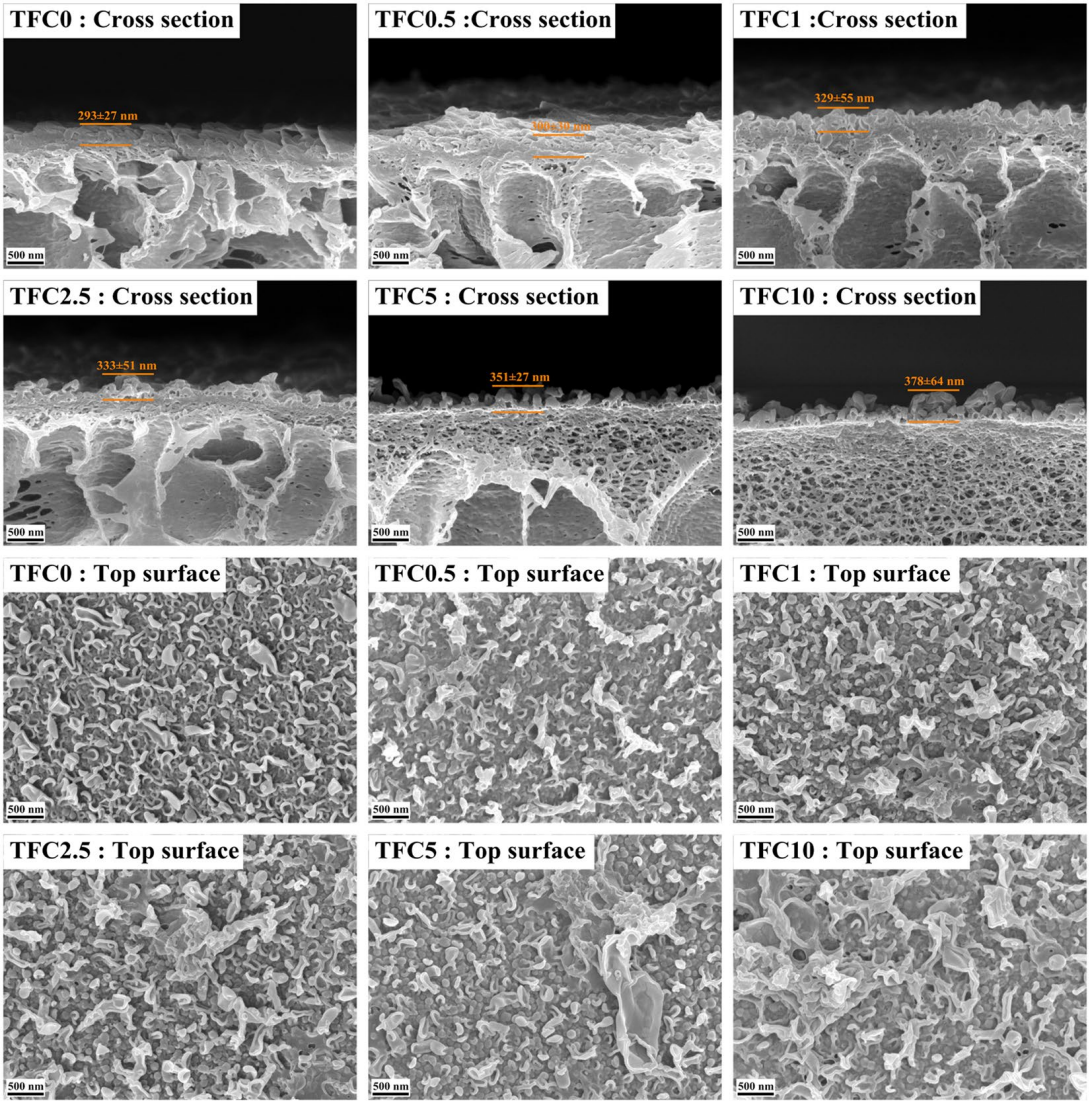
FESEM images of the active layers of different TFC membranes.

The polyamide active layer was synthesized on the substrates via an interfacial polymerization reaction between TMC and MPD at the oil-water interface. FESEM images (Fig. 3) were used to illustrate the morphological changes in the active layers of the TFC membranes. According to the top surface images, all the active layers of the TFC membranes exhibited a typical ridge-and-valley morphology. However, there are obvious differences between the neat PVC-based and SPSU/PVC-based TFC membranes. The top surface of TFC0 exhibited a smoother, nodular-like structure. The top surface of the SPSU/PVC based TFC membranes had rougher, grass-like structures, and more open top surface structures were observed as the SPSU blend ratio increased. The pore size difference may be the major factor resulting from this phenomenon. During the interfacial polymerization, MPD molecules migrated to the oil-water interface via diffusion and simple convection on substrates with smaller pore size. In contrast, this migration is dominated by the more vigorous Marangoni convection rather than diffusion and simple convection. The Marangoni convection results in a turbulent flow, enlarge the reaction contact area and may even push around, rotate, twist and bend the early-formed polyamide domains^8,38^. Hence, the polyamide active layer formed on the substrates with larger pores would exhibit a rougher and more open structure. Meanwhile, according to the images of the cross-sections, the thickness increased with the SPSU ratio. This phenomenon is probably because the SPSU-blended substrates with a higher porosity can hold more MPD solution to react with the TMC molecules. Consequently, the TFC membranes based higher SPSU ratio substrates exhibited thicker polyamide active layers^26^. In addition, since the substrates a higher SPSU ratio can hold more MPD solution, the water molecules could compete with the MPD to react with the TMC molecules, resulting in a less crosslinked polyamide structure. This theory was confirmed by the following XPS tests.

**Figure 4:**
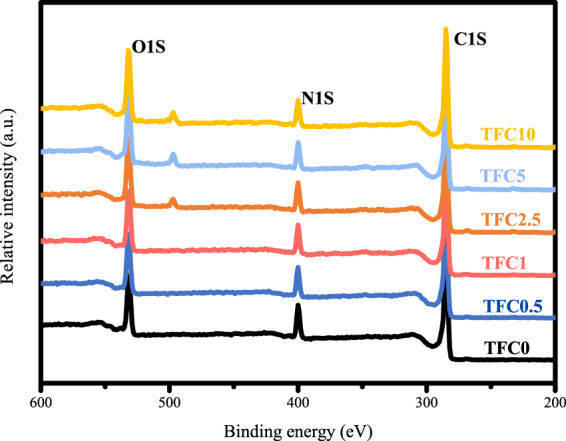
Low-resolution XPS spectra of the active layers in different TFC membranes.

**Figure 5:**
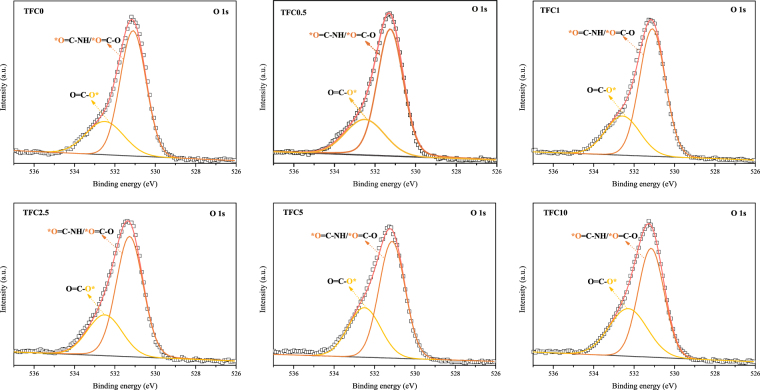
High-resolution XPS O 1 s spectra of the active layers in different TFC membranes.

The chemical composition of the polyamide active layer was analyzed by the low-resolution XPS tests, and the results are exhibited in Fig. 4 and Table 3. According to Table 3, the oxygen atom composition increased with the increase in the SPSU ratio, and the nitrogen atom composition showed the opposite trend, which led to an obviously increased O/N ratio. The increased O/N ratio illustrates that the crosslinking degree of the active layer declined with an increase in the SPSU ratio^39^. Furthermore, high-resolution XPS tests were carried out to determine changes in the functional groups on the active layer surfaces, especially carboxyl groups; the results are shown in Fig. 5 and Table 4. The curve of the O 1 s spectrum could be resolved into two peaks at binding energies of 531.1 and 532.5 eV, which represent two existing states of oxygen. As shown in Fig. 5, one is HN-C=O* and O-C=O* (OI, 531.1 eV), and the other is *O-C=O (OII, 532.5 eV). The intensity ratios of IOI/IOII can be used to estimate the reduction degree of the carboxyl groups^39^. According to Table 4, the ratio of I_OI_/I_OII_ decreased with increasing SPSU, which illustrates that more carboxyl groups were formed from the acyl chloride groups on the active layer.

**Table 3:** Surface elemental composition of the active layers in different TFC membranes.

Membranes	C	N	O	O/N
TFC0	73.31	12.09	14.60	1.21
TFC0.5	73.94	10.98	15.08	1.37
TFC1	73.37	10.76	15.87	1.47
TFC2.5	73.00	10.55	16.44	1.56
TFC5	72.20	9.94	17.87	1.80
TFC10	72.49	9.48	18.03	1.90

**Table 4:** Surface chemical composition of the active layers in different TFC membranes by XPS O 1 s spectral analysis.

Membranes	OI	OII	OI/OII
TFC0	53,204.92	20,627.32	2.58
TFC0.5	54,435.55	21,724.29	2.51
TFC1	55,050.29	22,332.47	2.47
TFC2.5	57,721.78	24,354.10	2.37
TFC5	57,969.02	30,565.92	1.90
TFC10	53,865.64	34,567.34	1.56

#### Intrinsic properties of the TFC membrane

Table 5 compares the intrinsic transport properties of the TFC membranes. The pure water permeability (*A*) exhibited an obvious augmentation after SPSU was introduced. Compared to the low *A* value of 0.67 LHM/bar for TFC0, the *A* value of the TFC2.5 showed a 231.34% improvement. Hence, with respect to the *A* value, SPSU played a considerable role in enhancing the TFC membrane performance. This improvement can be explained by the fact that the active layer became looser and less crosslinked after the SPSU was blended into the substrates. For same reason, the NaCl rejection rate (*R*) decreased with the increase of the SPSU blend ratio. However, according to the results, the *R* value decreased slightly from 96.01% for TFC0 to 95.12% for TFC2.5, and then decline obviously to 89.85%. Furthermore, the salt permeability (*B*) showed a more significant increase than *A* value. According to Eq. (5), the salt permeability (*B*) is positive correlation with the *A* value, and negative correlation with the *R* value. Hence, the *B* value should increase more significantly while *A* value increases and *R* decreases at the same time.

**Table 5:** Intrinsic properties of the TFC membranes.

Membranes	*A* ^a^		*R* (%)	*B*^b^ (×10^−8^ m/s)	*S*^c^ (μm)
	LMH/bar	×10^−12^ m/s Pa			
TFC0	0.67 ± 0.07	1.86 ± 0.21	96.01 ± 0.26	2.68 ± 1.74	2668 ± 147
TFC0.5	1.29 ± 0.13	3.58 ± 0.36	95.40 ± 0.33	6.03 ± 0.58	823 ± 50
TFC1	1.76 ± 0.18	4.89 ± 0.49	95.32 ± 0.32	8.40 ± 1.09	427 ± 29
TFC2.5	2.54 ± 0.22	7.06 ± 0.62	95.12 ± 0.96	12.64 ± 2.45	337 ± 19
TFC5	2.71 ± 0.10	7.53 ± 0.29	93.27 ± 1.06	19.10 ± 3.77	313 ± 7
TFC10	2.80 ± 0.26	7.78 ± 0.72	89.85 ± 0.43	30.85 ± 4.38	286 ± 16

^a^*A* values were measured in RO testing mode at 5.0 bar pressure with DI water as the feed solution.

^b^*B* values were measured in RO testing mode at 5.0 bar pressure with 2000 ppm NaCl as the feed solution.

^c^*S* values were measured under FO mode using 1 M NaCl as the draw solution and DI water as the feed solution.

The structure parameter (*S*) can be expressed as the diffusion distance for solutes to cross the substrate layer and is used as a metric to evaluate the ICP in the FO process^30^. Generally, a smaller *S* value indicates a lower level of ICP^9,40^. As shown in Table 3, the *S* value for TFC0 exhibited an extremely high value of 2668 μm, which indicates that the neat PVC substrate is not suitable for the preparation of a high-performance TFC FO membrane. However, the *S* values declined sharply after SPSU was introduced into the substrates. The *S* values decreased to 337 μm for TFC2.5, and 286 μm for TFC10 lastly.

### Performance of the TFC FO membranes

**Figure 6:**
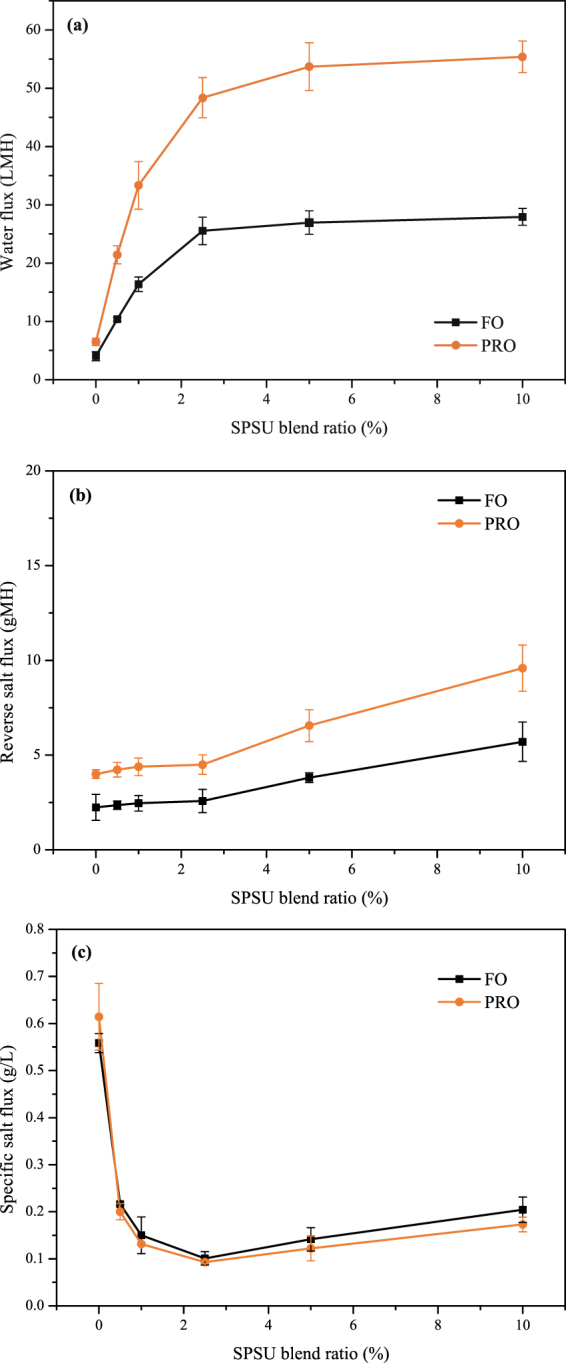
FO performance of the TFC FO membranes based on different substrates: (**a**) water flux, (**b**) reverse salt flux, and (**c**) specific salt flux.

The performance of the TFC FO membranes prepared by different substrates was assessed under both FO and PRO modes using 1 M NaCl as the draw solution and DI water as the feed solution. The water flux, reverse salt flux and specific salt flux are shown in Fig. 6.

Figure 6(a) shows the water flux of the TFC membranes prepared from different substrates. Consistent with most prior works^33,37,41^, higher water fluxes were observed under the PRO mode than the FO mode for all TFC membranes. This phenomenon can be explained by the fact that a more severe dilutive ICP would reduce the osmotic driving force across the FO membrane and decrease the water flux in the FO mode, while a slightly concentrative ICP occurs in the PRO mode^41^. After SPSU was introduced into the substrates, the water fluxes significantly improved. As it can be seen from Fig. 6(a), the water flux was remarkably improved from 4.02 LMH for TFC0 to 25.53 LMH for TFC2.5 under the FO mode, and from 6.50 LMH for TFC0 to 48.37 LMH for TFC0 under the PRO mode. This phenomenon can be attributed to the thinner and looser active layer and lower *S* value, which lead to a higher water permeability and lower ICP^40^. However, the improvement in the water flux was limited when the SPSU blend ratio was above 2.5%.

The reverse salt flux results under different modes are shown in Fig. 6(b). The trend of the reverse salt flux is consistent with that of the *R* data in the membrane intrinsic properties tests. The reverse salt fluxes slightly improved from 2.25 gMH for TFC0 to 2.57 gMH for TFC2.5 under the FO mode, and from 3.99 gMH for TFC0 to 4.50 gMH for TFC2.5 under the PRO mode. Then, the reverse salt fluxes obviously improved to 5.71 gMH for TFC10 under the FO mode, and to 9.59 gMH for TFC10 under the PRO mode. This phenomenon can be attributed to that the less crosslinked polyamide active layer would reducing the salt rejection efficiency and increasing the reverse salt flux^19^.

The specific salt flux (reverse salt flux/water flux ratio) is considered an explicit performance metric that can be used to assess the osmotic process efficiency and compare membrane performances of different membranes. A high-performance FO membrane requires a high water flux and low reverse salt flux; thus, a membrane with a low specific salt flux is preferred^30^. As can be seen in Fig. 6(c), the specific salt flux declined significantly after SPSU was introduced into the substrates and reached the minimum of 0.10/0.09 g/L (FO/PRO mode). Moreover, because of the increase in the reverse salt flux, the specific salt flux slightly increased when the SPSU blend ratio was above 2.5%.

**Figure 7:**
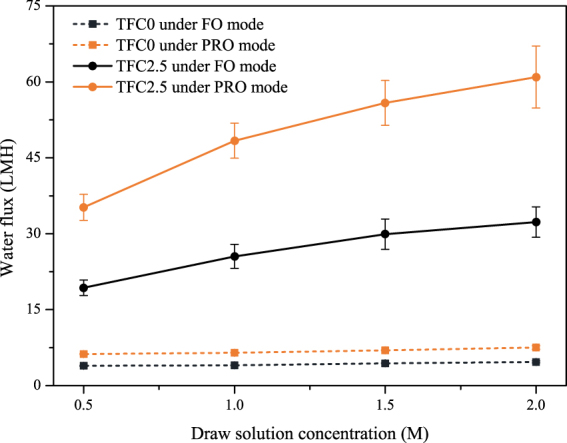
Water flux of TFC0 and TFC2.5 for various draw solution concentrations using DI water as the feed solution.

Figure 7 exhibits the water fluxes results for TFC0 and TFC2.5 under both FO and PRO modes as a function of the draw solution concentration. The overall water flux increased with the draw solution concentration. However, the water fluxes of TFC2.5 increased more significantly than that of TFC0. According to the data, the water flux of TFC2.5 increased 67.01%/73.12% from 0.5 M to 2.0 M NaCl draw solution under FO/PRO mode, while the water flux of TFC0 increased only 19.84%/21.07%. This significant difference between the neat PVC-based and SPSU/PVC-based TFC membranes could illustrate that the introduction of SPSU could obviously reduce the ICP during the FO process^27,37^.

### Performance comparison with other sulfonated materials based TFC membranes

**Figure 8:**
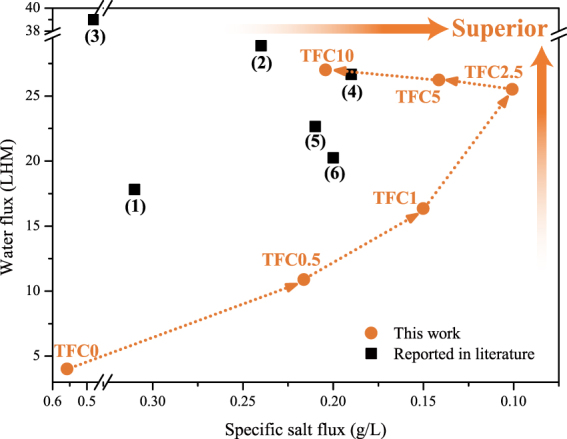
Comparison of the FO performances in this work with those of other sulfonated material-based TFC FO membranes reported in the literature. The serial numbers of the TFC membranes reported in the literature correspond to the NO. in Table 4.

It is believed that blending a sulfonated polymer with a backbone polymer to fabricate a substrate is a feasible strategy for improving the performance of a TFC FO membrane^9,25–28,33^. Table 6 and Fig. 8 compare the FO performances of the different sulfonated polymers-based TFC FO membranes in this work and those published in the literature. All of the TFC FO membranes were tested under the FO mode by using 1 M NaCl and DI water as the draw solution and feed solution. Compared to other sulfonated polymer based TFC membranes, the TFC2.5 with the lowest sulfonated blend ratio exhibited an excellent performance, while the other TFC membranes reported in the literature required blends above 12.5% sulfonated polymers (even 50%) to obtain the same-level water flux. Generally, sulfonated polymers are more expensive than the backbone polymers in material markets. A lower sulfonated blend ratio can reduce the manufacturing cost of fabrication the TFC membranes. Figure 6 plots both the specific salt flux and water flux of these TFC membranes. A superior performance for both the specific salt flux and water flux is presented in the top right corner. According to Fig. 8, the FO performance was significantly improved after SPSU was introduced into the substrates and was optimized at a sulfonated blend ratio of 2.5%. Moreover, the TFC2.5 exhibited a better performance than other sulfonated polymers-based TFC membranes in the reported literature.

**Table 6:** Comparison of the performances of the TFC membranes in this work and those reported in the literature.

NO.	Backbone polymer	Sulfonated polymer	Blend ratio	*J*_*W*_ (LMH)	*J*_*S*_ (gMH)	*J*_*S*_/*J*_*W*_ (g/L)	Reference
TFC0	PVC	—	0%	4.02	2.25	0.56	This work
TFC2.5	PVC	SPSU	2.5%	25.53	2.57	0.10	This work
TFC10	PVC	SPSU	10%	27.93	5.40	0.20	This work
(1)	PES	SPSU	12.5%	17.81	5.44	0.31	^25^
(2)	PSU	DSPAES	25%	28.87	6.82	0.24	^27^
(3)	PSU	SPSU	25%	39.00	18.90	0.48	^33^
(4)	PSU	SPPO	50%	26.67	5.18	0.19	^9^
(5)	PSU	SPEK	50%	22.65	4.73	0.21	^26^
(6)	PES	PES-co-SPPSU	50%	20.26	3.98	0.20	^28^

## Conclusion

In this work, a low-cost and high-performance TFC FO membrane was prepared using inexpensive PVC as the backbone substrate material and SPSU as the hydrophilic modifier. The FESEM study illustrated that the morphologies of the SPSU/PVC substrates were obviously looser and more porous than that of the neat PVC substrate. By increasing the ratio of SPSU and PVC, the porosity, PWP, surface hydrophilicity, and average pore size of the substrates significantly improved. Furthermore, it was observed that the polyamide active layer of the TFC membranes became rougher, looser and less crosslinked after SPSU was introduced. The *A* value obviously increased, and the *S* value dramatically declined. These results suggested that the introduction of SPSU is a feasible strategy to improve the performance and decrease the ICP of TFC membranes. Based on a comprehensive consideration of the properties of the FO membranes and the performance tests, it can be concluded that the SPSU blend ratio of xx% was the optimal blend ratio (e.g., the water flux of TFC2.5 reached xx/xx LMH under the FO/PRO mode using 1.0 M NaCl/DI water as the draw/feed solution, while the specific salt flux exhibited a low value of xx/xx g/L). The comparison studies determined that TFC2.5 exhibited the best FO performance with the lowest sulfonated blend ratio for sulfonated polymer-based TFC membranes. Hence, it is a feasible and low-cost fabrication approach for higher-performance TFC FO membranes using cheap PVC as the backbone material and low blend-ratio SPSU as the modifier material.
